# Experts' Encounters in Antenatal Diabetes Care: A Descriptive Study of Verbal Communication in Midwife-Led Consultations

**DOI:** 10.1155/2012/121360

**Published:** 2012-05-16

**Authors:** Christina Furskog Risa, Febe Friberg, Eva Lidén

**Affiliations:** ^1^Department of Education and Sports Science, Faculty of Arts and Education, University of Stavanger, 4036 Stavanger, Norway; ^2^Institute of Health and Care Sciences, Sahlgrenska Academy at Gothenburg University, 40530 Gothenburg, Sweden; ^3^Department of Health Studies, Faculty of Social Sciences, University of Stavanger, 4036 Stavanger, Norway

## Abstract

*Aim*. We regard consultations as cocreated communicatively by the parties involved. In this paper on verbal communication in midwife-led consultations, we consequently focus on the actual conversation taking place between the midwife and the pregnant woman with diabetes, especially on those sequences where the pregnant woman initiated a topic of concern in the conversation. *Methods*. This paper was undertaken in four hospital outpatient clinics in Norway. Ten antenatal consultations between midwives and pregnant women were audiotaped, transcribed to text, and analyzed using theme-oriented discourse analysis. Two communicative patterns were revealed: an expert's frame and a shared experts' frame. Within each frame, different communicative variations are presented. The topics women initiated in the conversations were (i) delivery, time and mode; (ii) previous birth experience; (iii) labor pain; and (iv) breast feeding, diabetes management, and fetal weight. *Conclusion*. Different ways of communicating seem to create different opportunities for the parties to share each other's perspectives. Adequate responses and a listening attitude as well as an ambiguous way of talking seem to open up for the pregnant women's perspectives. Further studies are needed to investigate the obstacles to, and premises for, providing midwifery care in a specialist outpatient setting.

## 1. Introduction

In line with the global increase in prediabetes and diabetes, a significant rise in the proportion of diabetes during pregnancy has been reported. Women with diabetes are regarded as a high-risk group with respect to maternal, fetal, and neonatal outcomes [1]. According to the Medical Birth Registry in Norway [2] reporting for the year 2008, diabetes was reported in 2.1% of all pregnancies. Diabetes type I (T1DM) and type II (T2DM) were diagnosed in 0.4% and 0.3%, respectively, of the pregnancies in 2009. Gestational diabetes mellitus (GDM) was recorded in 1.4% of pregnancies, a number that has doubled during the last decade.

In Norway, antenatal care for diabetes pregnancies is delivered in hospital outpatient clinics by multidisciplinary diabetes teams, in line with national guidelines for diabetes in pregnancy [3]. In this team, the midwife is responsible for continuity of follow-ups of the developing pregnancy. In addition, the midwife provides information and advice related to the consequences of living with diabetes and the expressed needs of the woman. Several studies point to the fact that midwifery consultations are perceived as valuable for the women [4, 5], but there is limited knowledge on how these consultations are carried out and performed in an antenatal diabetes care setting.

### 1.1. Previous Research on Pregnancy and Diabetes

There are numerous studies on the risk for perinatal outcomes related to pregnancy and diabetes from a medical perspective [1]. However, fewer studies have been conducted on women's perceptions of diabetes and pregnancy [6]. The research on diabetes in pregnancy could be divided into two groups: research of prepregnancy conditions in women with T1DM and T2DM, and research on GDM [7].

Women with T1DM have been reported to be more stressed and anxious compared to women in normal pregnancies [8]. During pregnancy, diabetes self-management is demanding. Feelings of guilt and blame for the unborn baby are prevalent. Berg [9] stresses the importance of the provider acting in a manner that promotes the woman's mastery of her condition instead of acting as a controller.

Women have reported the consultations with the professionals to be overshadowed by the diabetes diagnosis and several studies have revealed a lack of communicative topics related to normal pregnancy and maternity developmental tasks including focus on emotional needs [5, 10, 11]. Women with T1DM have also been reported to have a large need of access to reliable information during the whole childbearing period, not only in pregnancy [12].

### 1.2. Women with Gestational Diabetes

According to Sjögren et al. [13], pregnant women receiving a GDM diagnosis show great concerns about the baby's health as well as their own. In their study, women with GDM recalled having been being more worried during pregnancy compared to women without this pregnancy complication.

Persson et al. [14] describe women with GDM as experiencing a chaotic situation, one of being out of balance. They face different challenges to women who already have a diabetes diagnosis when entering pregnancy as they have to become confident with blood sugar monitoring procedures as well as adapting to dietary restrictions. Some of these women need to start administering insulin. According to Griffiths et al. [15], it is important to provide information in a timely manner once the diagnosis is set, as well as to tailor health advice to the actual woman's situation.

Regardless of the type of diabetes, women with diabetes in pregnancy are likely to have a higher risks of preeclampsia, a baby large for gestational age, shoulder dystocia, and Cesarean section compared to women with normal pregnancies [16, 17].

### 1.3. Provider Perspectives on Counseling Women with Diabetes in Pregnancy

There are a few studies of providers' perspectives of providing care for women with diabetes during pregnancy. Berg and Dahlberg [18], reporting on midwives working in hospital settings, describe them as struggling to balance the medical and normal perspectives, here the view of childbearing process as a normal life which also has to be supported, in their provision of midwifery care for women at high risk, including women with T1DM.

 In community primary health care settings, Persson et al. [19] found midwives counseling women with a GDM diagnosis as experiencing moral and ethical challenges in exerting control and providing the women with support as well as promoting lifestyle changes. Linell and Bredmar [20] discuss the moral implications of midwifery consultations in primary health care/community-based settings in talking about lifestyle-focused topics such as smoking, drinking, and sexually transmitted diseases and risk for malformations of the fetus.

Midwifery consultations for women with diabetes could therefore be regarded as *complex* consultations, during which the midwife encounters all types of women, regardless of parity and diagnosis, in a vulnerable period of their life. From the research, it is clear that women in diabetic pregnancies seem to have an extended need for emotional, appraisal, and informational support during their childbearing period including pregnancy [10, 21].

The professional midwife has an explicit responsibility to enable the pregnant women to be involved and participate in decisions of different kinds [22]. At the same time, the pregnant women must have the opportunity to express any concerns she has about the pregnancy, motherhood, and any other issues, concerns about life and living. These midwife-woman encounters could be considered as social processes where two experts meet, the woman as an expert on her life and living, and the midwife, as the professional expert in childbearing-related issues. Health care personnel have a special responsibility for ensuring that patients set their own agenda and as far as possible understand what is offered from a professional perspective [23].

Previous research about women with diabetic pregnancies examines either the perceptions and experiences of the women or the providers' perspectives, mainly through qualitative interviews. Less focus has been directed to the conversations taking place between midwives and women in consultations and practice.

Findings from our previous study [24] suggest that pregnant women to a lesser degree, compared to the midwives, initiated topics with concerns in the conversations. This indicates the relevance of describing consultations as cocreated communicatively by the parties. To improve clinical practice, we first need to illuminate and examine the taken-for-granted practice and behaviors in order to reflect on and challenge the provision of midwifery care. To gain such knowledge is important for midwives as well as other health care providers, who have a responsibility for promoting health and wellbeing in childbearing women.

Therefore, the aim of this study was to explore verbal communicative patterns in midwifery consultations, focusing on sequences where mothers initiated topics and concerns in the conversation.

The following research questions guided the analysis.

- *What topics and concerns were addressed/identified?*
- *Which variations in verbal communication were identified?*

## 2. Materials and Methods

### 2.1. Design

The study has an exploratory and interpretive design, based on the assumption that consultations between the midwife and the pregnant woman are socially constructed, situated, and relational phenomena. The midwife and pregnant woman contribute verbally and nonverbally to the understanding of the consultation situation [25–28]. This understanding of what happens during the consultation is produced and reproduced through social processes, with the language employed playing a significant role.

This study was conducted using a theme-oriented discursive approach, as described by Roberts and Sarangi [29], which means that we took a close look at how language constructs professional practice. We regard communication as not context-neutral; rather, ways of talking are formed and take place in a discourse space within a larger context, such as, in this case, societal expectations of midwifery consultations.

### 2.2. Settings

Five antenatal diabetes clinics at hospitals in urban areas of Norway were initially contacted about participation in the study, one of which declined. The study includes clinics at three university hospitals and one regional/local hospital. The diabetes team—an obstetrician, an endocrinologist, and a midwife—met weekly on a particular day at the clinics. The pregnant women were scheduled to meet the team every 14 days and sometimes weekly. The midwifery consultations varied from 12 to 45 minutes.

All consultation rooms were equipped with a desk, a computer and chairs, a scale, and equipment for measuring blood pressure. Some rooms had an examination bench where the midwife could conduct a physical examination of the pregnant woman. Some consultation rooms were also equipped with an ultrasound machine and a cardiotocograph (CTG) machine.

### 2.3. Participants and Recruitment Procedure

We invited 40 pregnant women to participate, asking them for permission to audio-record one of their consultations in the second or third trimester. The reason for selecting women at this stage of pregnancy was that we assumed that the midwife-mother relationship was well established by then. We also asked the women to take part in a postconsultation interview, which is not presented in this study. The inclusion criteria were having a diabetic condition and no other medical complication and being fluent in speaking and understanding Norwegian. The secretary at the clinic sent letters of request to women enrolled at the clinics during April and June 2008. Three women were given their letters by the midwife on the appointment day. Midwives working at the diabetes clinics were invited to participate by letter of request, in addition to receiving oral information about the study. None of the midwives declined; only ten of the pregnant women agreed to participate.

The ten pregnant women ranged in age from 28 to 45 years. Five were primiparous, two were expecting their second and third baby, and one woman was pregnant with her fourth baby. All had postsecondary education and were employed outside their home, although two were on part-time sick leave. One woman was accompanied to the consultation by her partner. He participated in the conversation to a minor degree. The six participating midwives had between 20 and 30 years' experience as midwives. All were female and ranged in age from 51 to 58 years. Two of the midwives were also diabetes specialists.

### 2.4. Data Collection

A pilot study was carried out by the first author (C. F. Risa) prior to the main study, using informal conversations with midwives (and in some clinics also with doctors) and observing them in their daily work.

This revealed that different tasks as well as time scheduled for the midwifery consultations varied between the clinics, which informed the author's understanding of the context surrounding the consultations. To capture the verbal conversation, a voice recorder was placed on the desk, and a microphone was fastened to the midwife's collar. Since the consultation rooms were small and the researcher did not want to interfere with the consultation, she then left the room and was not present during the consultation. Our data consists of ten audio-recorded consultations conducted at one of the scheduled appointments with participants in their second as well as third trimester (gestational week 26–36).

### 2.5. Ethical Considerations

This study was conducted in accordance with the Declaration of Helsinki [30] and was approved by the Norwegian Ethical Committee (no. 013/08). Informed consent was obtained from all the participants before participation. Midwives were instructed to stop the recording if needed or at the women's request. All participants were informed that they could withdraw from the study at any time without stating a reason or explanation why. The voice recordings were transcribed without disclosing the identity of the speaker and were kept from unauthorized parties. In Section 3, descriptions of the participants' medical situation as well as circumstances in care have been limited to secure confidentiality.

### 2.6. Data Analysis

The audio recordings were transcribed verbatim by the first author. The transcriptions were read and compared to the recordings several times, to both check for accuracy and get a sense of the whole and a sense of what to look for [31]. Results from a previous study in the project [32] (authors) have revealed that the midwives dominated the discursive *space*; that is, they did most of the talking and also initiated most topics. Therefore, to understand and describe in detail the language constructs in midwifery consultations, we closely examined the text, especially those sequences where the pregnant women initiated topics and concerns in the conversation. In those sequences, we identified features of direct and indirect ways of talking as well as ambiguities, which in the theme-oriented approach are referred to as the “focal themes of the analysis” [29]. This impression led us to take a closer look at Goffman [33] and his notion of “frames,” “footings,” and face-saving strategies in interactions.

As we understand Goffman, the term “frame” refers to how participants make use or sense of events as they construct those events. It refers to structures of expectations, in the situation. According to Goffman, a “footing” is the ways in which we position ourselves in relation to other persons by managing the production or reception of an utterance [34, page 129]. We understand a change in footing as utterances where the participants shift their “frame” of conversation and therefore the relations. Face-preserving strategies are described as strategies used to determine the degree to which it is possible to be implicit/indirect or explicit/direct in a conversation. Brown and Levinson, in developing Goffman's ideas further, named face-preserving strategies “politeness strategies” [35]. According to Brown and Levinson, indirect politeness strategies can be used while disagreeing with more powerful persons. A strong request could, for instance, be softened or mitigated by use of phrases such as “I think,” “You could,” or “You would.” In this paper, we refer to these strategies as “ambiguous ways of talking.”

In the analysis, we also investigate* metaphors *and* choice of words*, as well as paralinguistic features, such as pauses and accentuations in speech. In the quotes, pauses are marked with an ellipsis (…) and their length in seconds is given in brackets, for example, “[*silence 6 seconds*]”. Accentuations in speech are underlined.

## 3. Findings

### 3.1. Communicative Patterns

In our analysis, we have categorized instances where, during the consultation, the pregnant women initiated concerns into different “frames,” namely, (i) the professional expert's frame, and (ii) the shared experts' frame. In this section, we give examples of each. Within each frame, different communicative patterns are presented.

### 3.2. The Professional Expert's Frame

The professional expert's frame describes patterns of conversation mainly derived from the professional's point of departure. They are characterized by one-way communication—communication from the viewpoint of institutional procedures and medical knowledge. The women used implicit and explicit strategies to express their concerns and the midwives responded *in an explicit way*. The women's perspectives were revealed to a lesser degree. The topics addressed by the women were concerns about *delivery time and mode* and *breastfeeding*, which we here will present in detail.

The first communicative variation within the professional expert's frame is “procedural talk,” as seen in the following conversation on delivery-related issues, including sensations of labor onset. The second variation is called “persuasion talk,” here shown in a conversation where women's breastfeeding was in focus, especially previous breastfeeding experience. The third variation is termed steering talk. A steering talk took place when a mother signaled a need to talk about her former birth experience.

### 3.3. Procedural Talk

The following excerpt is an example of procedural talk. The midwife explains the procedures and routines of delivery. The woman is expecting her second child. Her first child was delivered by Cesarean section. In this sequence, we join the conversation as the woman raises her concern about the coming childbirth as an implicit question, a think-aloud question, or ambivalent statement, “I don't know.” In line 11, the midwife talks in an objective and distanced manner while the woman acts as a listener. We interpret the woman's laugh as a way to alleviate a situation which she perceives as threatening—that is, as a face-saving strategy. However, the midwife steers the consultation forward (line 20) and initiates a shift in “footing” by introducing a new topic, the baby's kicks. The baby's health is drawn into the conversation and keeps the conversation in a question-answer pattern, a typical feature of the professional expert's frame.

C1

1. W: I really don't know, I'm thinking if I'm not going to feel contractions as a vague pain in the back?
2. M: If you get contractions it is more than cramps in your back, then, sure it is.
3. W: Yes.
4. M: You can be sure about that.
5. M: But since you are a diabetic, you will never be going longer—maybe I'm going to shut this door here—since you are a diabetic you will never be going longer than—
6. W: No.
7. M: If you haven't started labor by yourself by the due date, you will get started.
8. W: Yes.
9. M: But since you were previously delivered by Cesarean, we have to use some other method to start you up. We have to consider that you had an operation in your uterus. So it may be a little difficult to start you up. Quite simply.
10. W: I have always been thinking … I'm hoping to let go this time.
11. M: Hm, well those who tried both, think when the birth starts spontaneously, it is better than to be started, they think that it's something with the body that has decided for the baby to come out.
12. W: Yes.
13. M: But in your case, we have your health to take into consideration, we will never let it go past the due date.
14. M: If you haven't started by the due date you will automatically be started.
15. W: Hmm.
16. W: Since you had an operation in your uterus, you have a uterus that is not quite so able as if you hadn't been operated and so we have to use other methods to start you. Well, you will be informed about this if and when it becomes relevant. One doesn't need to talk so much about it just now, but so you got some ideas anyway.
17. W: Hmm.
18. M: So, then it becomes exiting as you know that before the [due date] you will have the baby or at the latest on the [due date].
19. W: Latest [*laughs*].
20. M: [*Introduces new topic*.] Have you felt some more kicks [referring to fetal movements]?

As they talk, it seems that the midwife misses the woman's concern, as in line 10 the woman says, “I'm hoping to let go this time.” We do not know if the mother is referring to hope to “let go of” a Cesarean section or a vaginal birth. In answer, the midwife merely repeats what has been said earlier.

### 3.4. Persuasion Talk

In the following, a persuasion sequence is given to exemplify exhortative talk. The pregnant woman has had previous breastfeeding experience, which she is willing to share with the midwife. From the conversation, it appears that the midwife does not pick up on, or shows little interest in, the woman's preferences. The woman's pauses and “hmmm,” which we interpret as a noncommittal agreement, suggest that she does not agree with what the midwife says. Such face-saving strategies are used when disagreeing with a more powerful person. In line 9, we interpret the woman's response “I think” as an attempt to soften her disagreement while still holding on to her own experience.

C4

 [*The previous topic was former birth experiences.*]

1. W: Yeah … but don't know how it's going to work out with breastfeeding but that [*abrupt speech*] …
2. M: Yes, there are some that only breastfeed.
3. W: But the first time—
4. M: [*Interrupts.*] Yeah, but the first time is history, you have to forget … OK?
5. W: Yeah.
6. M: You have to take what comes. Don't think that it didn't work then—
7. W: But I do.
8. M: But you can think in a way, I would like to try and see, because it would save you a lot of work.
9. W: I think it was much easier to give by bottle [bottle feed].
10. M: But think of everything you will have to boil, keep in order, and prepare, while you are out, wherever you go, it's like [*laughs*] so if you get this working so it is,… and if you don't have all meals it saves a lot of hassle …
11. W: [*Continues to talk about former experiences.*] I remember awfully well how much of a struggle it was; I had to get up and latch and pump and …
12. M: No it's not like that but if everything is OK this is the best for your baby and you.
13. W: Hmm.

### 3.5. Steering Talk

This is another way of keeping the talk in a certain frame but here the midwife seems to negotiate with the woman about why she has to keep strict control of her blood sugar level. In the following excerpt, the mother signals a need to talk about her former birth experiences.

The use of the word “things” (line 5) directs the focus to the pregnant woman's physical health (by “things” is meant physical measurements, e.g., blood pressure, etc.) and the completing of the maternity record. The footing is the talk-aloud sequence (“let's see … we are going to see how it is with your things”) to lead the woman's attention back to the expert's frame. “Let's see” (italicized below for ease of reference) is therefore interpreted as a footing. The midwife uses it to cut the conversation short in order to keep to her schedule. In line 6, it seems that the woman has already come to understand that there is no time to talk today and she modifies her initial request as a way to “save the midwife's face.”

In line 10, the midwife calls up a different frame, by saying “just for a chat,” which may call up a different situation, where the woman may be encouraged talk about her situation with the midwife on a more equal footing. The last “let's see” is understood as a way of verbally signaling a change in “footing” to keep up with the “things,” that is, her tasks that have to be done.

C8

 [*They have been talking about previous deliveries.*]

1. M: Hmm, it was a vacuum and a forceps delivery but you can say, it was the first time and probably a bit of a case of your rupture then, hmm, it probably is, yeah.
2. W: Yeah, but I'm thinking, if I'm going to have a birth I need to go through this whole birth process thoroughly to know what happened.
3. M: Yes, there is for sure a possibility of that, yeah. When are you scheduled for the next appointment, is it about 2 weeks?
4. W: Yes, I don't know, it is usually every 2 weeks.
5. M: Yes, it becomes that, doesn't it, you know, *so let's see *… we are going to see how it is with your things.
6. W: Hmm, but for the time being there is no hurry.
7. M: A bit so it is time for that, it is as you know, it is so awfully hectic these days you are here, isn't it, but I was thinking we could arrange a pure midwifery consultation for you—
8. [*is interrupted by a colleague who is asking a question from the doorway*]
9. M:—so we could arrange that and go through and have a decent talk, but you should then visit [name] because it's she who's usually here those days [*confidential tone*].
10. W: It's actually the same—
11. M: Actually the same, OK, then we try to find a time before then and quite simply book you in for a day, other than the diabetes control day, just for a chat, but of course we'll check your blood pressure and all that but—
12. M: Yes.
13. M: I think we'll do that, won't we, so we'll have a lot of time so it wouldn't be like on top of everything else. Then, *let's see,* and then we attach this blood pressure thing … so I can go out and look at your urine … *let's see* … [*proceeds with the blood pressure measurement*].

### 3.6. The Shared Experts' Frame

The shared experts' frame describes a more two-way dialogue. The conversations that took place in this frame were composed of midwives reasoning from a medical viewpoint but at the same time taking account of the women's understanding and experiences. The women addressed their concerns both in an explicit and in an implicit way. However, the midwives were more prone to communicate in an ambiguous way in their responses and explanations. The following topics were introduced into the conversation by the women: (i) diabetes management and fetal weight, (ii) early signs of labor, and (iii) labor pain.

In the first communicative variation, “unpacking talk,” the midwife unravels, or “unpacks,” the woman's concern about diabetes management. The second variation is called “contextualization talk,” during which the midwife seems to reveal the woman's perceptions and expectations and transforms the information into contextual knowledge. Finally, the way in which the conversational dynamics can change during a conversation is illustrated by the “switch talk,” where the midwife introduces an episode of a shared experts' frame into an otherwise professional expert's frame.

### 3.7. Unpacking Talk 

In this sequence, we quote part of a conversation where, early in the consultation, a first-time mother is being greeted by the midwife who acknowledges the mother's belly. The mother's response is made impersonal and objective by the midwife's words, “We shouldn't expect the opposite, should we …?” The midwife seems to encourage the woman to voice any concerns by accentuating the pronoun *you*. This is to signify that she wants to hear about the woman herself and, briefly, shift the focus away from the baby. Over the next few sentences, the mother and midwife take a conversational journey with a lot of back and forth, assisted by the midwife's listening, comments, and questions as well as the use of continuers such as *hmm*,* yes*, and* uhhm*. These continuers seem to signal and call up a listening frame, *I want to hear more, keep on talking.* In this particular sequence, it takes about 36 lines before the mother shares her concerns about the baby's weight as a consequence of her blood sugar fluctuations. Note the hesitations and unfinished sentences which may indicate that she is approaching a sensitive topic. However, the possible resistance interpreted in this sequence could also be due to the fact that the mother did not really want to talk about this issue with the midwife. 

C5

1. M: I almost forgot this … it's been a long time since I last saw you, anyway it's been almost the whole summer, I wonder when we saw each other … [*looking in the maternity records*] well … it was in the last week of June … well, it's 2 months … now a lot has happened to you, your belly—
2. W: Yeah, it has grown.
3. M: Mightily.
4. W: Yes.
5. M: We shouldn't expect the opposite, should we, but how are you now?
6. W: In general very well, I feel the body is very heavy, I do that.
7. M: Yeah …
8. W: I keep on gaining …
9. M: Yeah …
10. W: I, and I have some nights that aren't so good.
11. M: Yeah … Because you have bad sleep?
12. W: I sleep very badly.
13. M: What do you do then?
14. W: I can't do anything except try to sleep.
15. M: What do you do then; do you get up or what?
16. W: Yes [*laughs*]. That's what I do.
17. M: Yeah.
18. W: The less sleep I have [the more] it affects my blood sugar; it changes when I can't sleep—
19. M: Yes … stress may well … make it rise, maybe?
20. W: Yes.
21. M: Do you sleep badly when … your husband isn't home, or?
22. W: No …
23. M: It doesn't have anything to do with that?
24. W: More like when I am more active the sleep becomes poor; more contractions—
25. M: Well—
26. W: Yeah, I sleep much worse.
27. M: So it's the contractions that wake you up?
28. W: Well, not wake me up but keep me awake.
29. M: So it's not that you fall asleep.
30. W: No, you know I have to watch my blood sugar at least every third hour—
31. M: [*Surprised tone*] You have?
32. W: I haven't slept through one night since …
33. [*Continues talking about diabetes management and tests.*]
34. M: When you think, what are you concerned about? What's in your head?
35. W: [*Silence 3 seconds.*] Blood sugar. [*Silence 3 seconds.*] I don't like the fluctuations; I find it [sic] very distressing.
36. M: You want control.
37. W: Yeah, yeah and I know … too, I think I sort of know how much this kid weighs; does he stress enormously those hours it [the blood sugar] goes high …

### 3.8. Contextualization Talk

In the following “contextualization talk,” a first-time mother-to-be is asking about the hospital routines during the early stages of labor. The midwife leaves her sentences unfinished, and there are delays and hesitations which result in an ambiguous way of talking; this is contrasted by the more direct and fact-oriented sequences in the expert's frame. The ambiguous talk, with its unfinished sentences, in this sequence may indicate the sensitive nature of diabetic pregnancies. Pregnant diabetic women are likely to have more interventions than women with normal pregnancies and may not have natural onset of labor. However, the midwife provides a context for the explanation that is understandable to the woman. 

C3

1. W: Is it something special that I as a diabetic need to think of when I think it [the birth] has started? Any routines, like I have to call earlier or something—or can I wait as long as others?
2. M: As long as you feel fine and your blood sugar's fine and as long as you in a way have control over it and eat and it doesn't lie too high or low, it must lie between 4 and 8—
3. W: I see. [Norwegian: *Jaja.*]
4. M: —and you feel fine and feel [fetal] movements then it's normal for you as for others; then it's really good.
5. W: I see. [Norwegian: *Jaja.*]
6. M: You do recognize kicks and movements like that while you also have contractions, yeah?
7. W: Yeah, OK.
8. M: Hope you will start by yourself so …
9. W: I hope so but you never know.
10. M: No, one can never know, one can hope because having the birth started is as if …
11. W: Yeah, that is probably what it is.
12. M: It's often more strenuous so it is often better when you start at home.
13. W: I see.
14. M: You can hang around at home and maybe you have moved into the new house?
15. Later on in the consultation, the woman inadvertently introduces her concerns in response to the midwife's direct question. In line 21, the midwife's utterance may be interpreted as indirect advice regarding what the woman should expect during birth. The woman seems to embrace the advice, as we interpret her responses of “I see,” and “Yeah, yeah …” as confirmations of the message.
16. [*Continuing to talk about practical issues.*]
17. M: Exactly, when you were attending the antenatal class did you get any more thoughts or were you thinking, was it something new that was revealed or something you …
18. W: Yes, it was some kind of scary. It didn't sound quite good with that epidural in my spine or near to, then—
19. M: Yes, it's not in the spine, it is—
20. W: I didn't like it and I hope to give birth as naturally as possible but I should maybe be thinking of having a bit of, hmm, pain relief because I can't bear to have it as if …
21. M: Hmm.
22. W: I'm not against it but haven't … I don't have to take it if I don't need to, that's what I think.
23. M: I think that's something you'll come to agree.
24. W: Yes, there and then.
25. M: Often it's given.
26. W: I see.
27. M: And if it stops and they see you are struggling and are very strained—
28. W: Yeah, yeah alright.
29. M: —and worn out, one would maybe …
30. W: Yeah, right, that's how I see it.
31. M: Yes, sometimes it is, when one can relax a little, your body becomes [*making an exhalation*] and then it can, all of a sudden, just open up when the bodily resistance in a way has gone.
32. W: Yeah yeah.
33. M: So then they may suggest, but no-one would do anything until you …
34. W: No no, it's not that I don't—[*interrupted/overlapping speech*]
35. M: It's not an exam.
36. W: No, and so I will choose the safest way.

### 3.9. Switch Talk

This excerpt serve as an example of how, during the talk, the midwife shifts her footing by interposing an episode of a shared experts' frame before returning to the expert's frame. The incomplete sentence in line 13 is metaphorically seen as a key which opens up for the woman's own ideas of her situation. In line 24, the midwife turns her attention to the CTG machine. The conversation is then continued on a question and answer basis in keeping with the expert's frame.

C1

1. M: Then I'm going to take this one [the CTG probe] away … and then it's smart to, while you are at home, you write down questions if you have something you wonder about, OK?
2. W: Yes.
3. M: It then becomes easier for you to remember when you come here.
4. W: Anyhow it's not so long since last time.
5. M: It's not, you are perfectly right.
6. W: But I'm on the Internet …
7. M: Hmm, it isn't always you find the best, hmm, answers on the Internet you know, we don't know, you can't guarantee the quality of what's been said on the Internet. You never know who's edited that.
8. W: No.
9. M: No.
10. W: But in a way, I'm quite calm …
11. M: Yeah, you look that way.
12. W: But it's usually when things tense up as—
13. M: It's maybe, maybe that you really don't know how this will turn out? If it's going to be the usual way or—?
14. W: I have always been like that; I can't really take on worrying myself.
15. M: No, that's smart though.
16. W: You never know really how it will work out.
17. M: That is absolutely smart … just about that [*unstrapping the CTG from the woman's belly*].
18. W: I think a little about I'm becoming a multipara [she was pregnant with her second child] when it comes to birthing.
19. M: Well, you are a second parous woman, but you are not multiparous in your body; in your birth canal you are like a primipara.
20. W: Yes.
21. M: Yes, since no baby has passed through your birth canal, you are regarded as a primipara. Since you haven't been at the beginning of a birth.
22. W: So I'm not really multiparous.
23. M: You are perfectly right; you are a primipara in your body and birth canal.
24. M: Here you see acceleration and there an acceleration [*looking on the CTG printout*] and it's having variations, great ups and downs, perfectly excellent as far as I can see anyway.

## 4. Discussion

The two patterns that emerged from the analysis reflect some strategies employed by midwives in responding to women's concerns, in an professional expert's frame or a shared experts' frame, using either a direct way of talking or an indirect way of talking. In this section, we discuss the findings within each frame.

The first excerpt in the expert's frame, “procedural talk,” describes a communication pattern that could be characterized by a transmission model of communication. It shows the midwife talking in a general and objective way while informing the woman about routines and procedures. In the second excerpt, “persuasion talk,” we suggest that the midwife takes a health promotive role. She argues from a professional stance and does not take the woman's own experience into account. It may seem that the midwife is expecting the woman to comply with recommendations within the breastfeeding discourse, as it is expected that women breastfeed their children, no matter what [36].

Similar patterns of relating to each other have been reported in a study of midwifery antenatal consultations in a primary health care setting. The authors [37] found five different patterns, three of which were regarded as basic patterns, named, in that study, the “respectful gardener and her developing plants,” the “propagandist teacher,” and the “steering inspector.” Our finding of the expert's frame may be in line with the pattern of the “steering inspector” where the midwife either missed or disregarded the woman's concerns and actual situation, and instead imposed her own norms and expected compliance with those norms.

The last excerpt from an expert's frame is a further example of “steering talk,” where the surrounding context is revealed in the conversation, as the midwife seems to explain the circumstances of midwifery consultations to the woman. We can ask if the midwife, with her choice of words and metaphors, is implicitly saying that talk is not the main agenda at these consultations. She refers to talk as something that happens in addition to the actual purpose of the consultation, the checkup. Alternatively, the word “pure” may be interpreted to mean that the current consultation is contaminated with medical issues and does not represent a proper midwifery consultation.

The pattern that the consultation takes may have consequences for how women perceive these consultations and comply with the expected behaviors and roles within the frame. We suspect that these women, some of them due to their background, have a history of encounters with health care professionals and may come to their consultation with a traditional provider-patient communication model in mind, which means the professional asks questions and the patient takes on a passive role, merely answering the questions.

As these consultations have a strong focus on monitoring tasks to enable early detection of complications, we suggest that there may be a risk that the woman comes to perceive herself as an object, a “container” for the unborn baby. On the other hand, on the basis of previous research we understand that women highly regard the *monitoring and surveillance aspects *of the regular biomedical assessments of the unborn baby's health [11, 38].

Because midwives are officially recognized as professional experts in childbearing issues, they should reflect on how they use and exercise their power in the consultation, especially as they seem to set the “tone” in the consultation, regarding which topics are given relevance in the consultation. On the other hand, it does seem that time limitations may necessitate a more tightly managed, expert consultation with focus on monitoring physical parameters at the expense of attention to emotional needs. As we have come to understand, as a consequence of the large increase in women with diabetes, primarily GDM, the diabetes team has to counsel more women within the same time frame and resources (personal communication). It may be that this expert's approach is a strategy to handle the workload and that without it; the individual consultation would delay the other team members' time schedule.

The “steering talk” sequence could possibly indicate such a conflict, as the midwife seems to explain and justify her need to keep up with her institutional demands at the expense of meeting the woman's need to talk. This may frame the midwife as an institutional representative. According to Hunter [39], midwives who do to not work in accordance with their ideal or philosophy may experience less work satisfaction as well as a variety of negative emotions, such as frustration, anxiety, and anger.

We also found that the frequent use of the pronoun *we *by the midwife in the procedural talk could be interpreted in line with the midwife's face work strategies. The recommendations do not stem from her; she is replicating the institution's voice [35].

The communication pattern in the shared experts' frame has a more two-way, dialogic approach where the midwife sets a listening context, as well as providing the woman with an opportunity to tell her story. It also seems that the point of departure for the conversation is the woman's actual situation; from there, the conversation continues, with an interweaving between the woman's personal knowledge and the midwife's professional and personal knowledge. From a pedagogical perspective, we can understand this in terms of how general information is transformed and contextualized to the individual pregnant woman, to be used in the forthcoming birth.

The midwife's listening attitude, with use of continuers such as *ahh *and* mmm*, seems to encourage the woman to open up about her concerns, rather than simply ask open questions. This is interesting because in contemporary health care, open questions are regarded as important tools in facilitating the expression of mothers' (and patients') views, symptoms, and concerns. However, studies, for example, by Suchman et al. [40], report that patients seldom explicitly reveal concerns, and that instead, they offer clues and statements from their life situation, which might be associated with an emotion. The provider often misses these cues, so a listening attitude from the professional may lead to a direct expression of the patient's emotional concerns.

Consequently, we suggest that it is insufficient to routinely ask open questions and lay the responsibility on the woman to voice her concerns explicitly and directly. In the example of the “switch talk,” the woman is being advised to collect and write down her questions and ask them at the next consultation. The beginning of this excerpt may mirror this consultation as an arena for information transfer, where knowledge is constructed by the parties and where both parties get to know something new.

Ambiguous talk was found in the shared experts' frame, and it was characterized by pauses and unfinished sentences, which suggest communicative caution. According to Linell and Bredmar [20], this is a way of informing the pregnant woman while aiming not to cause anxiety, a face-saving strategy in line in Goffmanian terms [33]. Similar patterns of ambiguous talk have been identified in studies where counseling touched lifestyle issues which may have moral implications, such as in the human immunodeficiency virus (HIV)/acquired immunodeficiency syndrome (AIDS) counseling context [41] and also in nurse-diabetic counseling encounters [42].

In this study, ambiguous ways of talking were revealed in topics regarding some general sources of uncertainty related to childbirth: the when, how, and outcome of the birth, as well as pain in birthing [43]. Ambiguous ways of talking may be a strategy for the midwife to handle information of a delicate nature, since aggregated to large groups, women in diabetic pregnancies are more prone to have more inductions, instrumental deliveries, and deliveries by Cesarean section compared to women in normal pregnancies and therefore may to a lesser degree come to experience normal birthing. The ambiguous way of talking, by being implicit and tentative, seems to function as an invitation from the midwife to the pregnant mother to have an input. Similar findings have been reported in a study of family therapy, where ambiguous ways of talking were denoted as reciprocal editing [44] of the ongoing talk.

### 4.1. Methodological Considerations

We found it challenging to demarcate the sequences in ongoing conversations, as the conversations had an evolving character; what one said influenced what the other said in a spiral fashion [45]. Therefore, some of the illustrated excerpts are long and still have to be regarded as “snap shots” from a unique and ongoing, whole consultation.

This study is based on transcriptions of voice recordings from consultations and some methodological considerations apply. We fully agree that transcripts are not an objective representation of a conversation; they are regarded as a theoretical contextual construction of reality [46]. The analytical perspective we used in this study is based on a linguistic sociological approach; a different analytical focus may well have led to different findings. We have taken into consideration the fact that transcripts can never reproduce all aspects of speech or the exchange between the individuals nor illuminate the whole of the participants' intended meanings, experiences, or intentions.

In order to get close to the data, the first author collected as well as transcribed the audiotapes into texts which were read and revised several times. A further consideration is that what is heard in the consultations depends on the hearer. The hearer in this case, the first author, is a nurse-midwife experienced in antenatal consultations in primary health care settings. This background was an advantage in the process of understanding the midwives' ways of working as well as their choice of vocabulary and phrases. The first authors discussed and reflected on the interpretations during the process. Due to the limited extent of this study, we have not presented the original Norwegian versions of the excerpts. This could have given the reader some information on the structure and semantics of the original language and may have allowed readers to make their own interpretations.

The advantage of “slowing down” an activity or talk by analyzing it at a microlevel is that it provides an opportunity to identify how participants process talk through a conversation. In our study, this led to an understanding of the complexity of midwifery consultations as well as raising questions as to other contextual situational factors which may have influenced the communication at a microlevel, such as the time available, the initial and subsequent contact with the actual midwife, and the incidence of the “knock on the door” interruptions by other professionals during the consultation [47, 48].

Individuals invited to participate in research do not need to state their reason for declining to take part, so we do not know the reasons for the low participation rate in this study. Voice-recording a consultation could possibly be regarded as intrusive and this may explain the low number of participants. Since communication is more than just verbal speech, the use of a video recorder may have captured some aspects of the interaction that were missed in the voice recording. However, the data collected were rich in detail and served the purpose of addressing the research questions in these consultations. In a larger material, further categories and patterns may have emerged.

One methodological question that needs to be raised is this: would the same interpretations as ours, the professional expert's and shared experts' frames, have evolved in consultations with other pregnant women in a different context? If so, would the patterns have been equally, more, or less expert-oriented?

The findings may not be transferable to all antenatal diabetes consultations in Norway or other countries that may have different health care. We suggest that the findings may provide insight into midwifery consultations beyond the particular settings and may also be useful for other health care professionals.

### 4.2. Conclusion and Implications for Practice

Different ways of communicating create different prerequisites for the mother's perspective to unfold as well as different opportunities for the parties to share each other's perspectives. From a professional perspective, providing adequate responses as well as creating space for a narrative approach with a listening attitude can be important for the mother's concerns and experiences to be revealed in the consultations. Professionals need to consider and discuss which behavior can best reach women, especially mothers in vulnerable situations such as high-risk pregnancies, and in which roles they can best assist them in their need to know and understand.

It is also important to address the premises of, and obstacles to, providing midwifery in a midwifery setting. The findings in this study may serve as a basis for further research aimed at broadening our understanding of the communicational dynamics in antenatal diabetes consultations.
